# Crystal structure of [1-(3-eth­oxy-2-oxido­benzyl­idene-κ*O* ^2^)-4-phenyl­thio­semicarbazidato-κ^2^ *N* ^1^,*S*](tri­phenylphosphane-κ*P*)nickel(II)

**DOI:** 10.1107/S2056989015021660

**Published:** 2015-11-21

**Authors:** B. Karpagam, G. Chakkaravarthi, G. Rajagopal

**Affiliations:** a Department of Chemistry, St.Michael College of Engineering & Technology, Kalayarkoil 630 551, India; b Department of Physics, CPCL Polytechnic College, Chennai 600 068, India; c PG & Research Department of Chemistry, Chikkanna Government Arts College, Tiruppur 641 602, India

**Keywords:** crystal structure, nickel(II), thio­semicarbazones, hydrogen bonding

## Abstract

In the title complex, [Ni(C_16_H_15_N_3_O_2_S)(C_18_H_15_P)], the Ni^II^ atom has a distorted tetra­hedral coordination geometry, comprised of N, S, O and P atoms of the tridentate thiosemicarbazide ligand and the P atom of the triphenylphosphane ligand. The benzene ring makes a dihedral angle of 53.08 (11)° with the phenyl ring of the phenyl­thio­semicarbazide moiety and dihedral angles of 73.69 (11), 20.38 (11) and 71.30 (11)° with the phenyl rings of tri­phenyl­phosphane ligand. A pair of N—H⋯N hydrogen bonds generates an *R*_2_^2^(8) ring graph-set motif. The eth­oxy group is disordered over two positions, with site occupancies of 0.631 (9) and 0.369 (9). The mol­ecular structure is stabilized by a weak intra­molecular C—H⋯O hydrogen bond. In the crystal, weak N—H⋯N and C—H⋯π inter­actions connect the mol­ecules, forming a three-dimensional network.

## Related literature   

For biological activities of thio­semicarbazones and their transition metal complexes, see: Hu *et al.* (2006 ▸); Banerjee *et al.* (2011 ▸); Pitucha *et al.* (2010 ▸). For reported similar structures, see: Islam *et al.* (2014 ▸); Zhang *et al.* (2004 ▸).
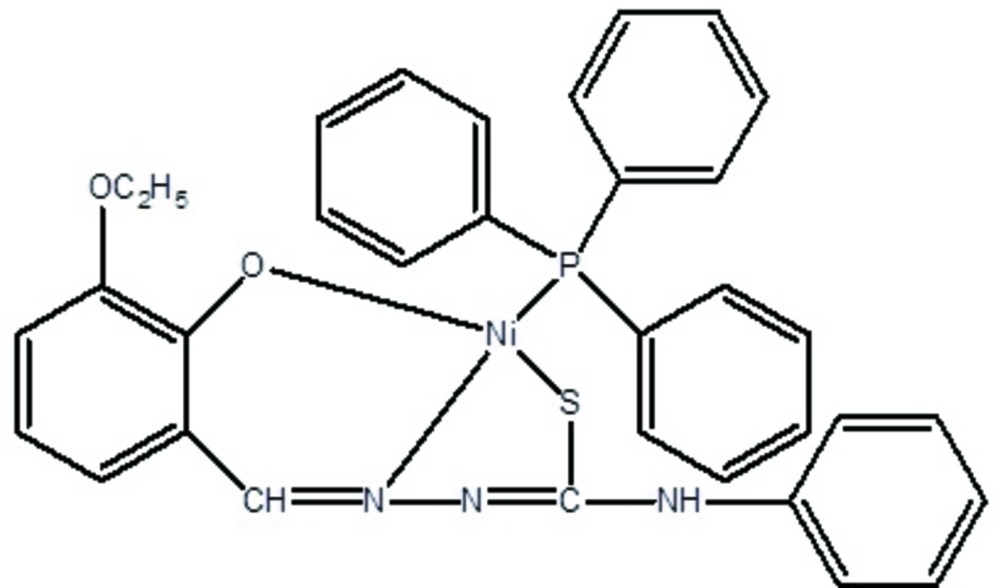


## Experimental   

### Crystal data  

[Ni(C_16_H_15_N_3_O_2_S)(C_18_H_15_P)]
*M* *_r_* = 634.35
Triclinic, 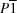

*a* = 9.7290 (2) Å
*b* = 12.9770 (3) Å
*c* = 14.0120 (2) Å
α = 62.958 (1)°
β = 73.756 (2)°
γ = 71.654 (1)°
*V* = 1475.91 (5) Å^3^
*Z* = 2
Mo *K*α radiation
μ = 0.82 mm^−1^
*T* = 295 K
0.28 × 0.24 × 0.20 mm

### Data collection  

Bruker Kappa APEXII CCD diffractometer
Absorption correction: multi-scan (*SADABS*; Sheldrick, 1996 ▸) *T*_min_ = 0.803, *T*_max_ = 0.853
32169 measured reflections
7289 independent reflections
6002 reflections with *I* > 2σ(*I*)
*R*_int_ = 0.030

### Refinement  

*R*[*F* ^2^ > 2σ(*F* ^2^)] = 0.030
*wR*(*F* ^2^) = 0.081
*S* = 1.03
7289 reflections
411 parameters
7 restraints
H atoms treated by a mixture of independent and constrained refinement
Δρ_max_ = 0.30 e Å^−3^
Δρ_min_ = −0.25 e Å^−3^

### 

Data collection: *APEX2* (Bruker, 2004 ▸); cell refinement: *SAINT* (Bruker, 2004 ▸); data reduction: *SAINT*; program(s) used to solve structure: *SHELXS97* (Sheldrick, 2008 ▸); program(s) used to refine structure: *SHELXL97* (Sheldrick, 2008 ▸); molecular graphics: *PLATON* (Spek, 2009 ▸); software used to prepare material for publication: *SHELXL97*.

## Supplementary Material

Crystal structure: contains datablock(s) global, I. DOI: 10.1107/S2056989015021660/ff2143sup1.cif

Structure factors: contains datablock(s) I. DOI: 10.1107/S2056989015021660/ff2143Isup2.hkl

**Fig. 1.**
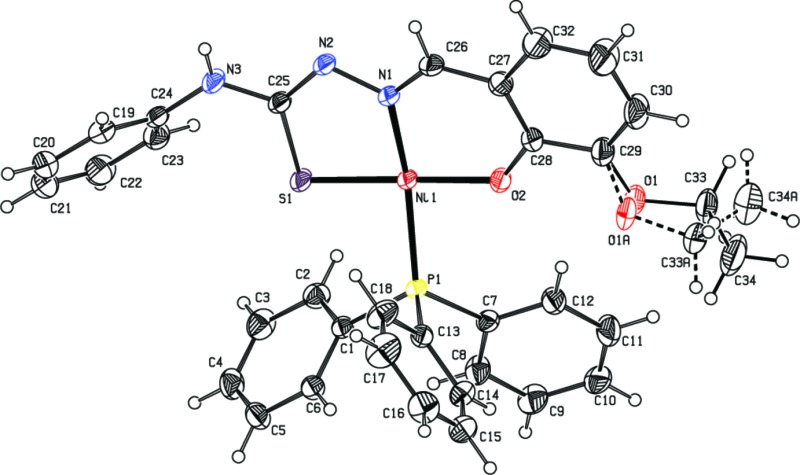
The molecular structure of (I), with atom labels and 30% probability displacement ellipsoids for non-H atoms.

**Fig. 2.**
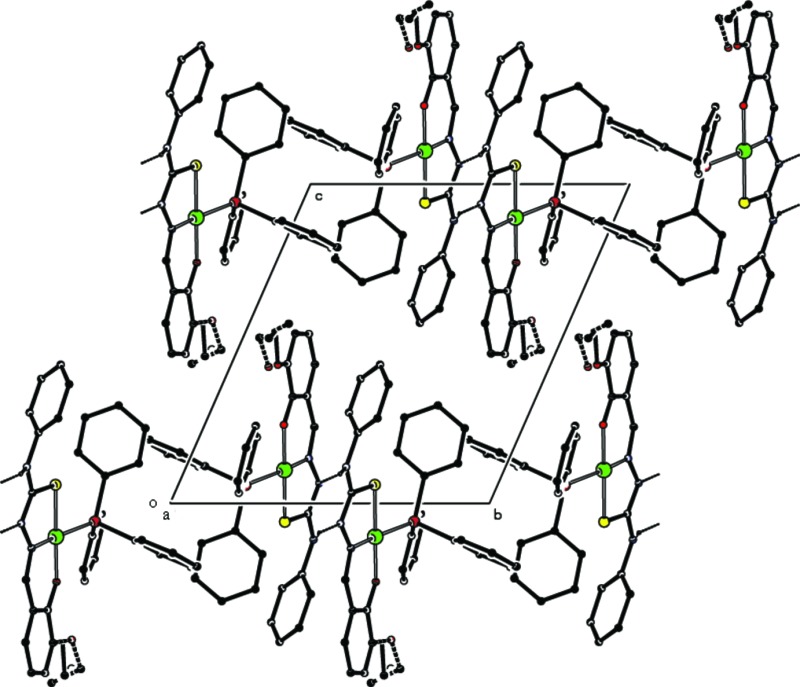
The crystal packing of the title compound, viewed along the a axis. The hydrogen bonds are shown as dashed lines (see Table 1). H atoms not involved in these interactions have been omitted for clarity.

**Fig. 3.**
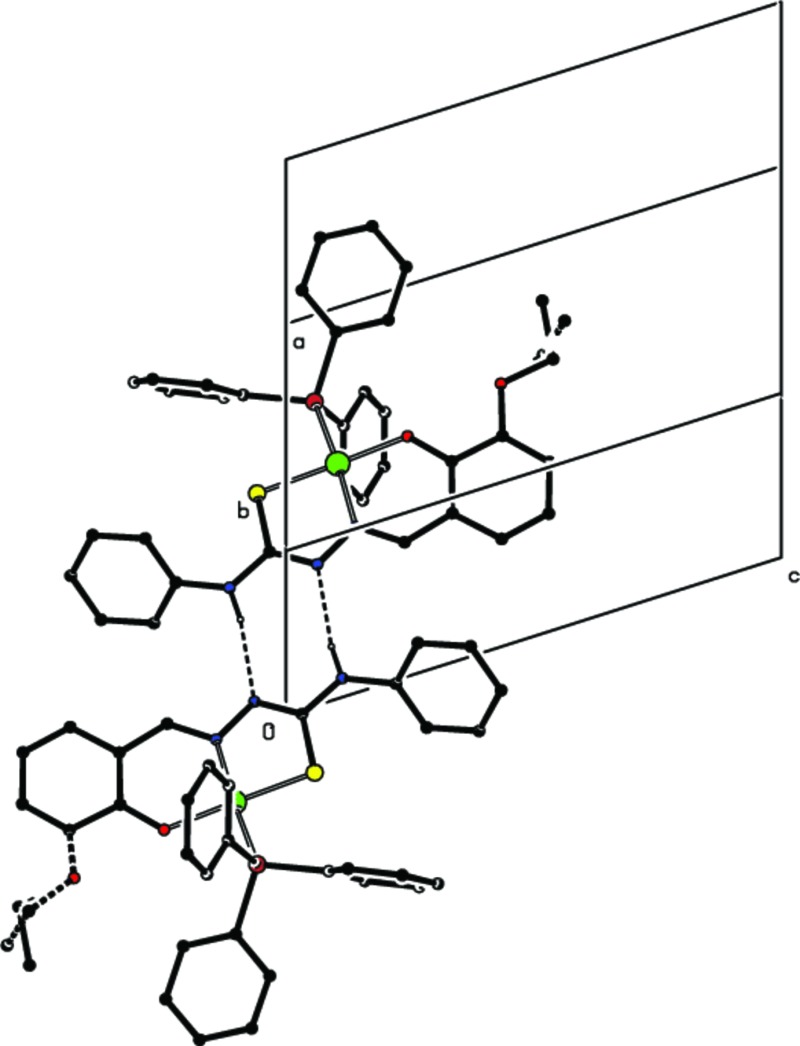
The partial crystal packing of the title compound, showing the ring set-motif viewed along the b axis. The hydrogen bonds are shown as dashed lines.

. DOI: 10.1107/S2056989015021660/ff2143fig1.tif
The mol­ecular structure of (I), with atom labels and 30% probability displacement ellipsoids for non-H atoms.

a . DOI: 10.1107/S2056989015021660/ff2143fig2.tif
The crystal packing of the title compound, viewed along the *a* axis. The hydrogen bonds are shown as dashed lines (see Table 1). H atoms not involved in these inter­actions have been omitted for clarity.

b . DOI: 10.1107/S2056989015021660/ff2143fig3.tif
The partial crystal packing of the title compound, showing the ring set-motif viewed along the *b* axis. The hydrogen bonds are shown as dashed lines.

CCDC reference: 964626

Additional supporting information:  crystallographic information; 3D view; checkCIF report

## Figures and Tables

**Table 1:** Hydrogen-bond geometry (Å, °) *Cg*7 is the centroid of the C27–C32 ring.

*D*—H⋯*A*	*D*—H	H⋯*A*	*D*⋯*A*	*D*—H⋯*A*
C12—H12⋯O1	0.93	2.46	3.314 (7)	154
C12—H12⋯O2	0.93	2.35	3.113 (2)	139
N3—H3*A*⋯N2^i^	0.87 (1)	2.22 (1)	3.0811 (19)	170 (2)
C33*A*—H33*D*⋯*Cg*7^ii^	0.97	2.79	3.279 (10)	112

Symmetry codes: (i) 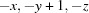; (ii) 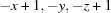.

## supplementary crystallographic information

### S1. Comment

Thiosemicarbazones and their transition metal complexes have revealed wide spectrum of activities such as anticancer (Hu *et al.*, 2006), anti-HIV (Banerjee *et al.*, 2011) and antitubercular (Pitucha *et al.*, 2010). We herein, report the crystal structure of the title compound (I), (Fig. 1). In the complex, the Ni1—S1, Ni1–N1, Ni1—P1 and Ni1—O2 bond distances are of 2.1355 (4), 1.8766 (12), 2.2291 (4) and 1.8676 (10)Å, respectively. These geometric parameters of the title compound are comparable to the reported structures (Islam, *et al.*, 2014; Zhang, *et al.*, 2004) and literature values.

The ethoxy group is disordered over two positions, with site occupancies of 0.631 (9) and 0.369 (9). The benzene ring (C27—C32) make the dihedral angle of 53.08 (11)° with the phenyl ring (C19—C24) of phenylthiosemicarbazide moiety. The dihedral angles between the benzene ring (C27—C32) and the phenyl rings (C1—C6), (C7—C12) and (C13—C18) of triphenyl phosphine moiety are 73.69 (11), 20.38 (11) and 71.30 (11)°, respectively. The intermolecular N3-H3A···N2 hydrogen bond generates R_2_^2^(8) ring-set motif.

The molecular structure is stabilized by a weak intramolecular C—H···O hydrogen bonds (Table 1) and the crystal structure is controlled by weak intermolecular N—H···N and C—H···π (Fig.2 & Table 1) interactions to form a three dimensional network.

### S2. Experimental

About 218 mg of the metal nickel triphenyl phosphine was dissolved in 5 ml of ethanol and the ligand 100 mg was dissolved in 3 ml of dichloromethane. Then the mixture was refluxed for 3 to 4 h in cool ice bath condition, since dichlomethane has a very low boiling point. The red colour solution was allowed to stand for about 5 days at room temperature. After this period of time, the resulting dark-red solids were collected by filtration, washed with 10 ml on n-hexane and dried *in vacuo* over anhydrous CaCl_2_. A single red colour crystal suitable for the X-ray diffraction was obtained by slow evaporation of a solution in acetonitrile.

### S3. Refinement

H atoms were positioned geometrically and refined using riding model with C—H = 0.93 Å and *U*_iso_(H) = 1.2Ueq(C) for CH, C—H = 0.97 Å and *U*_iso_(H) = 1.2Ueq(C) for CH_2_ and C—H = 0.96 Å and *U*_iso_(H) = 1.5Ueq(C) for CH_3_. H atom N atom is fixed from Fourier map and refined freely with distance restraint 0.88 (1) Å. The bond distances C29—O1, C29—O1A, O1—C33, and O1A—C33A were restraint to 1.40 (1) Å and the bond distances C33—C34 and C33A—C34A distances were restraint to 1.55 (1) Å with *DFIX* command in *SHELXL97* (Sheldrick, 2008).

### Crystal data

**Table:**

[Ni(C_16_H_15_N_3_O_2_S)(C_18_H_15_P)]	*Z* = 2
*M**_r_* = 634.35	*F*(000) = 660
Triclinic, *P*1	*D*_x_ = 1.427 Mg m^−^^3^
Hall symbol: -P 1	Mo *K*α radiation, λ = 0.71073 Å
*a* = 9.7290 (2) Å	Cell parameters from 2018 reflections
*b* = 12.9770 (3) Å	θ = 2.2–28.3°
*c* = 14.0120 (2) Å	µ = 0.82 mm^−^^1^
α = 62.958 (1)°	*T* = 295 K
β = 73.756 (2)°	Block, red
γ = 71.654 (1)°	0.28 × 0.24 × 0.20 mm
*V* = 1475.91 (5) Å^3^	

### Data collection

**Table:**

Bruker Kappa APEXII CCD diffractometer	7289 independent reflections
Radiation source: fine-focus sealed tube	6002 reflections with *I* > 2σ(*I*)
Graphite monochromator	*R*_int_ = 0.030
ω and φ scan	θ_max_ = 28.3°, θ_min_ = 2.2°
Absorption correction: multi-scan (*SADABS*; Sheldrick, 1996)	*h* = −12→12
*T*_min_ = 0.803, *T*_max_ = 0.853	*k* = −17→17
32169 measured reflections	*l* = −18→18

### Refinement

**Table:**

Refinement on *F*^2^	Primary atom site location: structure-invariant direct methods
Least-squares matrix: full	Secondary atom site location: difference Fourier map
*R*[*F*^2^ > 2σ(*F*^2^)] = 0.030	Hydrogen site location: inferred from neighbouring sites
*wR*(*F*^2^) = 0.081	H atoms treated by a mixture of independent and constrained refinement
*S* = 1.03	*w* = 1/[σ^2^(*F*_o_^2^) + (0.0392*P*)^2^ + 0.3532*P*] where *P* = (*F*_o_^2^ + 2*F*_c_^2^)/3
7289 reflections	(Δ/σ)_max_ < 0.001
411 parameters	Δρ_max_ = 0.30 e Å^−^^3^
7 restraints	Δρ_min_ = −0.25 e Å^−^^3^

### Special details

**Table:**

Geometry. All esds (except the esd in the dihedral angle between two l.s. planes) are estimated using the full covariance matrix. The cell esds are taken into account individually in the estimation of esds in distances, angles and torsion angles; correlations between esds in cell parameters are only used when they are defined by crystal symmetry. An approximate (isotropic) treatment of cell esds is used for estimating esds involving l.s. planes.
Refinement. Refinement of F^2^ against ALL reflections. The weighted R-factor wR and goodness of fit S are based on F^2^, conventional R-factors R are based on F, with F set to zero for negative F^2^. The threshold expression of F^2^ > 2sigma(F^2^) is used only for calculating R-factors(gt) etc. and is not relevant to the choice of reflections for refinement. R-factors based on F^2^ are statistically about twice as large as those based on F, and R- factors based on ALL data will be even larger.

### Fractional atomic coordinates and isotropic or equivalent isotropic displacement parameters (Å^2^)

**Table:**

	*x*	*y*	*z*	*U*_iso_*/*U*_eq_	Occ. (<1)
C1	0.75160 (16)	0.24179 (14)	−0.08641 (12)	0.0334 (3)	
C2	0.75713 (19)	0.35814 (16)	−0.15930 (14)	0.0447 (4)	
H2	0.7284	0.4181	−0.1342	0.054*	
C3	0.8053 (2)	0.38465 (19)	−0.26897 (15)	0.0558 (5)	
H3	0.8074	0.4626	−0.3174	0.067*	
C4	0.8499 (2)	0.2962 (2)	−0.30648 (15)	0.0571 (5)	
H4	0.8817	0.3143	−0.3802	0.068*	
C5	0.8475 (2)	0.18102 (19)	−0.23501 (15)	0.0517 (5)	
H5	0.8792	0.1211	−0.2604	0.062*	
C6	0.79814 (18)	0.15360 (15)	−0.12535 (13)	0.0400 (4)	
H6	0.7962	0.0754	−0.0775	0.048*	
C7	0.85493 (17)	0.19942 (14)	0.10147 (13)	0.0356 (3)	
C8	0.98860 (19)	0.20072 (18)	0.03254 (15)	0.0494 (4)	
H8	0.9925	0.2149	−0.0394	0.059*	
C9	1.1160 (2)	0.18122 (19)	0.06923 (18)	0.0564 (5)	
H9	1.2049	0.1821	0.0221	0.068*	
C10	1.1116 (2)	0.16066 (18)	0.17405 (18)	0.0556 (5)	
H10	1.1977	0.1462	0.1991	0.067*	
C11	0.9801 (2)	0.1613 (2)	0.24270 (19)	0.0672 (6)	
H11	0.9772	0.1484	0.3141	0.081*	
C12	0.8509 (2)	0.1808 (2)	0.20701 (16)	0.0551 (5)	
H12	0.7620	0.1813	0.2542	0.066*	
C13	0.66787 (17)	0.05795 (14)	0.11644 (12)	0.0343 (3)	
C14	0.77810 (19)	−0.03890 (15)	0.15956 (14)	0.0432 (4)	
H14	0.8666	−0.0271	0.1615	0.052*	
C15	0.7578 (2)	−0.15257 (16)	0.19954 (15)	0.0526 (5)	
H15	0.8328	−0.2169	0.2278	0.063*	
C16	0.6282 (3)	−0.17131 (18)	0.19790 (17)	0.0588 (5)	
H16	0.6145	−0.2481	0.2256	0.071*	
C17	0.5187 (3)	−0.0763 (2)	0.1552 (2)	0.0688 (6)	
H17	0.4307	−0.0888	0.1532	0.083*	
C18	0.5376 (2)	0.03834 (17)	0.11517 (18)	0.0549 (5)	
H18	0.4620	0.1023	0.0873	0.066*	
C19	0.1463 (2)	0.46798 (16)	−0.26655 (15)	0.0473 (4)	
H19	0.0689	0.4312	−0.2248	0.057*	
C20	0.1925 (3)	0.4802 (2)	−0.37291 (18)	0.0632 (6)	
H20	0.1456	0.4523	−0.4028	0.076*	
C21	0.3073 (3)	0.5336 (2)	−0.43442 (17)	0.0679 (6)	
H21	0.3388	0.5416	−0.5060	0.081*	
C22	0.3756 (2)	0.5751 (2)	−0.39001 (16)	0.0631 (6)	
H22	0.4543	0.6104	−0.4316	0.076*	
C23	0.3288 (2)	0.56516 (17)	−0.28433 (14)	0.0491 (4)	
H23	0.3739	0.5954	−0.2553	0.059*	
C24	0.21437 (17)	0.50998 (14)	−0.22197 (13)	0.0386 (4)	
C25	0.24291 (16)	0.44610 (14)	−0.03268 (12)	0.0359 (3)	
C26	0.20003 (18)	0.34640 (15)	0.23944 (13)	0.0424 (4)	
H26	0.0987	0.3739	0.2478	0.051*	
C27	0.2624 (2)	0.28071 (16)	0.33618 (13)	0.0454 (4)	
C28	0.41423 (18)	0.23648 (15)	0.33456 (12)	0.0390 (4)	
C29	0.4640 (2)	0.17511 (18)	0.43723 (14)	0.0511 (4)	
C30	0.3662 (3)	0.1579 (2)	0.53196 (16)	0.0761 (7)	
H30	0.4005	0.1163	0.5980	0.091*	
C31	0.2168 (3)	0.2014 (3)	0.53072 (17)	0.0930 (10)	
H31	0.1518	0.1890	0.5958	0.112*	
C32	0.1649 (2)	0.2618 (2)	0.43560 (16)	0.0752 (7)	
H32	0.0645	0.2910	0.4355	0.090*	
N1	0.26909 (13)	0.37212 (11)	0.14090 (10)	0.0336 (3)	
N2	0.17568 (14)	0.43889 (12)	0.06343 (10)	0.0379 (3)	
N3	0.16198 (15)	0.49892 (14)	−0.11384 (11)	0.0455 (4)	
O2	0.50937 (12)	0.24802 (10)	0.24580 (8)	0.0406 (3)	
P1	0.68938 (4)	0.21048 (3)	0.05755 (3)	0.03082 (9)	
Ni1	0.46961 (2)	0.312000 (17)	0.104547 (14)	0.03154 (7)	
S1	0.42853 (4)	0.38465 (4)	−0.05808 (3)	0.04352 (11)	
O1	0.6109 (6)	0.1463 (8)	0.4341 (6)	0.0517 (16)	0.631 (9)
C33	0.6533 (5)	0.0936 (6)	0.5392 (4)	0.0681 (16)	0.631 (9)
H33A	0.6287	0.0165	0.5813	0.082*	0.631 (9)
H33B	0.6046	0.1438	0.5786	0.082*	0.631 (9)
C34	0.8162 (6)	0.0818 (6)	0.5175 (4)	0.107 (2)	0.631 (9)
H34A	0.8521	0.0475	0.5851	0.161*	0.631 (9)
H34B	0.8385	0.1587	0.4747	0.161*	0.631 (9)
H34C	0.8624	0.0316	0.4787	0.161*	0.631 (9)
O1A	0.6128 (11)	0.1203 (14)	0.4277 (10)	0.057 (3)	0.369 (9)
C33A	0.6898 (11)	0.0516 (7)	0.5191 (7)	0.060 (2)	0.369 (9)
H33C	0.7794	0.0018	0.4978	0.072*	0.369 (9)
H33D	0.6296	−0.0003	0.5770	0.072*	0.369 (9)
C34A	0.7290 (13)	0.1242 (7)	0.5628 (7)	0.087 (3)	0.369 (9)
H34D	0.7741	0.0722	0.6261	0.130*	0.369 (9)
H34E	0.6416	0.1761	0.5817	0.130*	0.369 (9)
H34F	0.7962	0.1705	0.5083	0.130*	0.369 (9)
H3A	0.0681 (11)	0.5085 (18)	−0.0919 (16)	0.057 (6)*	

### Atomic displacement parameters (Å^2^)

**Table:**

	*U*^11^	*U*^22^	*U*^33^	*U*^12^	*U*^13^	*U*^23^
C1	0.0238 (7)	0.0430 (8)	0.0337 (7)	−0.0043 (6)	−0.0051 (6)	−0.0172 (6)
C2	0.0395 (9)	0.0455 (9)	0.0439 (9)	−0.0078 (7)	−0.0038 (7)	−0.0164 (7)
C3	0.0497 (11)	0.0589 (11)	0.0420 (10)	−0.0141 (9)	−0.0041 (8)	−0.0066 (8)
C4	0.0474 (11)	0.0840 (14)	0.0335 (9)	−0.0116 (10)	−0.0029 (8)	−0.0222 (9)
C5	0.0455 (10)	0.0719 (13)	0.0458 (10)	−0.0077 (9)	−0.0050 (8)	−0.0353 (10)
C6	0.0343 (8)	0.0480 (9)	0.0399 (8)	−0.0059 (7)	−0.0067 (7)	−0.0212 (7)
C7	0.0288 (8)	0.0389 (8)	0.0429 (8)	−0.0033 (6)	−0.0097 (6)	−0.0200 (7)
C8	0.0319 (9)	0.0725 (12)	0.0460 (10)	−0.0156 (8)	−0.0056 (7)	−0.0235 (9)
C9	0.0309 (9)	0.0746 (13)	0.0663 (12)	−0.0147 (9)	−0.0067 (8)	−0.0290 (11)
C10	0.0381 (10)	0.0664 (12)	0.0773 (14)	−0.0041 (9)	−0.0240 (9)	−0.0378 (11)
C11	0.0471 (12)	0.1075 (18)	0.0641 (13)	−0.0034 (11)	−0.0207 (10)	−0.0510 (13)
C12	0.0348 (9)	0.0864 (14)	0.0531 (11)	−0.0015 (9)	−0.0105 (8)	−0.0412 (11)
C13	0.0309 (8)	0.0397 (8)	0.0312 (7)	−0.0064 (6)	−0.0040 (6)	−0.0148 (6)
C14	0.0361 (9)	0.0445 (9)	0.0463 (9)	−0.0049 (7)	−0.0116 (7)	−0.0156 (7)
C15	0.0587 (12)	0.0414 (9)	0.0512 (10)	−0.0034 (8)	−0.0156 (9)	−0.0142 (8)
C16	0.0711 (14)	0.0466 (10)	0.0579 (12)	−0.0218 (10)	−0.0081 (10)	−0.0158 (9)
C17	0.0556 (13)	0.0652 (13)	0.0933 (17)	−0.0274 (11)	−0.0178 (12)	−0.0259 (12)
C18	0.0357 (10)	0.0527 (10)	0.0757 (13)	−0.0089 (8)	−0.0180 (9)	−0.0206 (10)
C19	0.0393 (9)	0.0474 (9)	0.0520 (10)	0.0008 (7)	−0.0124 (8)	−0.0212 (8)
C20	0.0630 (14)	0.0726 (14)	0.0646 (13)	0.0086 (11)	−0.0259 (11)	−0.0430 (11)
C21	0.0651 (14)	0.0838 (15)	0.0405 (10)	0.0083 (12)	−0.0080 (10)	−0.0293 (11)
C22	0.0523 (12)	0.0723 (14)	0.0430 (10)	−0.0078 (10)	0.0011 (9)	−0.0138 (10)
C23	0.0444 (10)	0.0543 (10)	0.0421 (9)	−0.0076 (8)	−0.0074 (8)	−0.0157 (8)
C24	0.0307 (8)	0.0403 (8)	0.0354 (8)	0.0055 (6)	−0.0098 (6)	−0.0133 (6)
C25	0.0272 (7)	0.0390 (8)	0.0362 (8)	0.0001 (6)	−0.0079 (6)	−0.0139 (6)
C26	0.0307 (8)	0.0497 (9)	0.0385 (8)	0.0036 (7)	−0.0039 (6)	−0.0199 (7)
C27	0.0404 (9)	0.0537 (10)	0.0322 (8)	−0.0009 (7)	−0.0029 (7)	−0.0167 (7)
C28	0.0400 (9)	0.0438 (9)	0.0316 (8)	−0.0042 (7)	−0.0070 (6)	−0.0163 (7)
C29	0.0499 (11)	0.0627 (11)	0.0359 (9)	−0.0055 (9)	−0.0121 (8)	−0.0175 (8)
C30	0.0683 (15)	0.1077 (19)	0.0312 (9)	−0.0042 (13)	−0.0108 (9)	−0.0184 (11)
C31	0.0641 (15)	0.142 (3)	0.0324 (10)	−0.0011 (15)	0.0044 (10)	−0.0219 (13)
C32	0.0480 (12)	0.1078 (19)	0.0403 (10)	0.0043 (12)	0.0015 (9)	−0.0239 (11)
N1	0.0260 (6)	0.0373 (6)	0.0337 (6)	0.0022 (5)	−0.0076 (5)	−0.0155 (5)
N2	0.0270 (6)	0.0443 (7)	0.0340 (7)	0.0042 (5)	−0.0079 (5)	−0.0149 (6)
N3	0.0255 (7)	0.0641 (9)	0.0348 (7)	0.0038 (6)	−0.0075 (6)	−0.0171 (7)
O2	0.0304 (6)	0.0562 (7)	0.0309 (5)	−0.0018 (5)	−0.0072 (4)	−0.0176 (5)
P1	0.02285 (18)	0.0374 (2)	0.03196 (19)	−0.00183 (14)	−0.00561 (14)	−0.01614 (15)
Ni1	0.02392 (10)	0.03883 (11)	0.02941 (10)	0.00097 (7)	−0.00641 (7)	−0.01555 (8)
S1	0.02623 (19)	0.0627 (3)	0.03223 (19)	0.00587 (17)	−0.00726 (15)	−0.01987 (18)
O1	0.0459 (19)	0.071 (4)	0.0370 (16)	−0.0052 (15)	−0.0166 (13)	−0.0200 (17)
C33	0.063 (3)	0.095 (4)	0.040 (2)	−0.002 (3)	−0.024 (2)	−0.022 (2)
C34	0.071 (3)	0.164 (6)	0.070 (3)	0.011 (3)	−0.042 (3)	−0.039 (3)
O1A	0.060 (4)	0.067 (6)	0.044 (3)	−0.006 (3)	−0.031 (3)	−0.016 (3)
C33A	0.067 (5)	0.063 (5)	0.043 (4)	0.001 (3)	−0.025 (4)	−0.015 (3)
C34A	0.098 (8)	0.093 (6)	0.071 (5)	−0.023 (5)	−0.038 (5)	−0.020 (4)

### Geometric parameters (Å, º)

**Table:**

C1—C6	1.384 (2)	C22—C23	1.379 (3)
C1—C2	1.394 (2)	C22—H22	0.9300
C1—P1	1.8240 (15)	C23—C24	1.380 (2)
C2—C3	1.385 (3)	C23—H23	0.9300
C2—H2	0.9300	C24—N3	1.411 (2)
C3—C4	1.375 (3)	C25—N2	1.297 (2)
C3—H3	0.9300	C25—N3	1.355 (2)
C4—C5	1.373 (3)	C25—S1	1.7367 (15)
C4—H4	0.9300	C26—N1	1.293 (2)
C5—C6	1.384 (2)	C26—C27	1.420 (2)
C5—H5	0.9300	C26—H26	0.9300
C6—H6	0.9300	C27—C28	1.404 (2)
C7—C12	1.377 (2)	C27—C32	1.413 (3)
C7—C8	1.386 (2)	C28—O2	1.3038 (19)
C7—P1	1.8227 (16)	C28—C29	1.430 (2)
C8—C9	1.380 (2)	C29—O1	1.351 (6)
C8—H8	0.9300	C29—C30	1.370 (3)
C9—C10	1.359 (3)	C29—O1A	1.396 (9)
C9—H9	0.9300	C30—C31	1.386 (3)
C10—C11	1.369 (3)	C30—H30	0.9300
C10—H10	0.9300	C31—C32	1.350 (3)
C11—C12	1.389 (3)	C31—H31	0.9300
C11—H11	0.9300	C32—H32	0.9300
C12—H12	0.9300	N1—N2	1.3986 (17)
C13—C18	1.375 (2)	N1—Ni1	1.8766 (12)
C13—C14	1.385 (2)	N3—H3A	0.867 (9)
C13—P1	1.8226 (16)	O2—Ni1	1.8676 (10)
C14—C15	1.378 (3)	P1—Ni1	2.2291 (4)
C14—H14	0.9300	Ni1—S1	2.1355 (4)
C15—C16	1.365 (3)	O1—C33	1.433 (6)
C15—H15	0.9300	C33—C34	1.499 (6)
C16—C17	1.368 (3)	C33—H33A	0.9700
C16—H16	0.9300	C33—H33B	0.9700
C17—C18	1.384 (3)	C34—H34A	0.9600
C17—H17	0.9300	C34—H34B	0.9600
C18—H18	0.9300	C34—H34C	0.9600
C19—C24	1.376 (2)	O1A—C33A	1.440 (8)
C19—C20	1.380 (3)	C33A—C34A	1.514 (8)
C19—H19	0.9300	C33A—H33C	0.9700
C20—C21	1.370 (3)	C33A—H33D	0.9700
C20—H20	0.9300	C34A—H34D	0.9600
C21—C22	1.371 (3)	C34A—H34E	0.9600
C21—H21	0.9300	C34A—H34F	0.9600
			
C6—C1—C2	118.85 (15)	N2—C25—S1	122.28 (12)
C6—C1—P1	122.34 (12)	N3—C25—S1	119.85 (12)
C2—C1—P1	118.77 (12)	N1—C26—C27	126.96 (15)
C3—C2—C1	120.20 (17)	N1—C26—H26	116.5
C3—C2—H2	119.9	C27—C26—H26	116.5
C1—C2—H2	119.9	C28—C27—C32	120.72 (17)
C4—C3—C2	120.22 (18)	C28—C27—C26	122.12 (15)
C4—C3—H3	119.9	C32—C27—C26	117.16 (17)
C2—C3—H3	119.9	O2—C28—C27	123.68 (14)
C5—C4—C3	119.97 (17)	O2—C28—C29	119.47 (15)
C5—C4—H4	120.0	C27—C28—C29	116.85 (15)
C3—C4—H4	120.0	O1—C29—C30	122.8 (3)
C4—C5—C6	120.34 (18)	C30—C29—O1A	126.1 (6)
C4—C5—H5	119.8	O1—C29—C28	116.2 (3)
C6—C5—H5	119.8	C30—C29—C28	120.74 (18)
C1—C6—C5	120.41 (17)	O1A—C29—C28	112.1 (5)
C1—C6—H6	119.8	C29—C30—C31	120.92 (19)
C5—C6—H6	119.8	C29—C30—H30	119.5
C12—C7—C8	118.72 (16)	C31—C30—H30	119.5
C12—C7—P1	120.03 (13)	C32—C31—C30	120.4 (2)
C8—C7—P1	121.08 (13)	C32—C31—H31	119.8
C9—C8—C7	120.85 (17)	C30—C31—H31	119.8
C9—C8—H8	119.6	C31—C32—C27	120.4 (2)
C7—C8—H8	119.6	C31—C32—H32	119.8
C10—C9—C8	120.13 (18)	C27—C32—H32	119.8
C10—C9—H9	119.9	C26—N1—N2	112.89 (13)
C8—C9—H9	119.9	C26—N1—Ni1	124.00 (11)
C9—C10—C11	119.76 (18)	N2—N1—Ni1	122.73 (10)
C9—C10—H10	120.1	C25—N2—N1	111.59 (12)
C11—C10—H10	120.1	C25—N3—C24	125.84 (14)
C10—C11—C12	120.80 (19)	C25—N3—H3A	114.2 (14)
C10—C11—H11	119.6	C24—N3—H3A	117.7 (14)
C12—C11—H11	119.6	C28—O2—Ni1	127.12 (10)
C7—C12—C11	119.71 (18)	C13—P1—C7	104.13 (7)
C7—C12—H12	120.1	C13—P1—C1	103.46 (7)
C11—C12—H12	120.1	C7—P1—C1	102.25 (7)
C18—C13—C14	118.68 (16)	C13—P1—Ni1	104.28 (5)
C18—C13—P1	118.34 (13)	C7—P1—Ni1	122.93 (5)
C14—C13—P1	122.96 (12)	C1—P1—Ni1	117.52 (5)
C15—C14—C13	120.54 (17)	O2—Ni1—N1	94.98 (5)
C15—C14—H14	119.7	O2—Ni1—S1	178.91 (4)
C13—C14—H14	119.7	N1—Ni1—S1	86.08 (4)
C16—C15—C14	120.43 (18)	O2—Ni1—P1	88.98 (4)
C16—C15—H15	119.8	N1—Ni1—P1	166.97 (4)
C14—C15—H15	119.8	S1—Ni1—P1	90.029 (15)
C15—C16—C17	119.51 (18)	C25—S1—Ni1	97.19 (5)
C15—C16—H16	120.2	C29—O1—C33	113.5 (5)
C17—C16—H16	120.2	O1—C33—C34	105.1 (4)
C16—C17—C18	120.6 (2)	O1—C33—H33A	110.7
C16—C17—H17	119.7	C34—C33—H33A	110.7
C18—C17—H17	119.7	O1—C33—H33B	110.7
C13—C18—C17	120.25 (18)	C34—C33—H33B	110.7
C13—C18—H18	119.9	H33A—C33—H33B	108.8
C17—C18—H18	119.9	C33—C34—H34A	109.5
C24—C19—C20	120.34 (19)	C33—C34—H34B	109.5
C24—C19—H19	119.8	H34A—C34—H34B	109.5
C20—C19—H19	119.8	C33—C34—H34C	109.5
C21—C20—C19	120.0 (2)	H34A—C34—H34C	109.5
C21—C20—H20	120.0	H34B—C34—H34C	109.5
C19—C20—H20	120.0	C29—O1A—C33A	123.3 (10)
C20—C21—C22	119.76 (19)	O1A—C33A—C34A	114.7 (9)
C20—C21—H21	120.1	O1A—C33A—H33C	108.6
C22—C21—H21	120.1	C34A—C33A—H33C	108.6
C21—C22—C23	120.7 (2)	O1A—C33A—H33D	108.6
C21—C22—H22	119.6	C34A—C33A—H33D	108.6
C23—C22—H22	119.6	H33C—C33A—H33D	107.6
C22—C23—C24	119.45 (19)	C33A—C34A—H34D	109.5
C22—C23—H23	120.3	C33A—C34A—H34E	109.5
C24—C23—H23	120.3	H34D—C34A—H34E	109.5
C19—C24—C23	119.70 (16)	C33A—C34A—H34F	109.5
C19—C24—N3	118.93 (16)	H34D—C34A—H34F	109.5
C23—C24—N3	121.33 (16)	H34E—C34A—H34F	109.5
N2—C25—N3	117.79 (14)		
			
C6—C1—C2—C3	1.5 (3)	C26—N1—N2—C25	−168.96 (15)
P1—C1—C2—C3	179.34 (14)	Ni1—N1—N2—C25	4.26 (19)
C1—C2—C3—C4	−0.9 (3)	N2—C25—N3—C24	−176.89 (16)
C2—C3—C4—C5	−0.3 (3)	S1—C25—N3—C24	0.0 (3)
C3—C4—C5—C6	1.0 (3)	C19—C24—N3—C25	124.94 (19)
C2—C1—C6—C5	−0.8 (2)	C23—C24—N3—C25	−57.1 (3)
P1—C1—C6—C5	−178.58 (13)	C27—C28—O2—Ni1	−5.5 (3)
C4—C5—C6—C1	−0.4 (3)	C29—C28—O2—Ni1	174.13 (13)
C12—C7—C8—C9	1.4 (3)	C18—C13—P1—C7	168.08 (15)
P1—C7—C8—C9	−173.88 (16)	C14—C13—P1—C7	−13.76 (16)
C7—C8—C9—C10	−0.2 (3)	C18—C13—P1—C1	−85.35 (15)
C8—C9—C10—C11	−1.1 (3)	C14—C13—P1—C1	92.81 (14)
C9—C10—C11—C12	1.0 (4)	C18—C13—P1—Ni1	38.09 (15)
C8—C7—C12—C11	−1.4 (3)	C14—C13—P1—Ni1	−143.74 (13)
P1—C7—C12—C11	173.92 (17)	C12—C7—P1—C13	−75.24 (16)
C10—C11—C12—C7	0.2 (4)	C8—C7—P1—C13	99.97 (15)
C18—C13—C14—C15	0.5 (3)	C12—C7—P1—C1	177.28 (15)
P1—C13—C14—C15	−177.64 (14)	C8—C7—P1—C1	−7.50 (16)
C13—C14—C15—C16	−0.5 (3)	C12—C7—P1—Ni1	42.54 (17)
C14—C15—C16—C17	0.6 (3)	C8—C7—P1—Ni1	−142.24 (13)
C15—C16—C17—C18	−0.8 (4)	C6—C1—P1—C13	−12.23 (15)
C14—C13—C18—C17	−0.7 (3)	C2—C1—P1—C13	169.99 (13)
P1—C13—C18—C17	177.52 (17)	C6—C1—P1—C7	95.76 (14)
C16—C17—C18—C13	0.9 (4)	C2—C1—P1—C7	−82.02 (14)
C24—C19—C20—C21	0.5 (3)	C6—C1—P1—Ni1	−126.48 (12)
C19—C20—C21—C22	−0.3 (3)	C2—C1—P1—Ni1	55.74 (14)
C20—C21—C22—C23	−0.8 (3)	C28—O2—Ni1—N1	10.45 (14)
C21—C22—C23—C24	1.7 (3)	C28—O2—Ni1—P1	−157.11 (14)
C20—C19—C24—C23	0.4 (3)	C26—N1—Ni1—O2	−11.00 (15)
C20—C19—C24—N3	178.31 (16)	N2—N1—Ni1—O2	176.53 (12)
C22—C23—C24—C19	−1.4 (3)	C26—N1—Ni1—S1	169.23 (14)
C22—C23—C24—N3	−179.34 (17)	N2—N1—Ni1—S1	−3.23 (11)
N1—C26—C27—C28	1.4 (3)	C26—N1—Ni1—P1	96.3 (2)
N1—C26—C27—C32	−179.0 (2)	N2—N1—Ni1—P1	−76.2 (2)
C32—C27—C28—O2	178.0 (2)	C13—P1—Ni1—O2	74.61 (6)
C26—C27—C28—O2	−2.4 (3)	C7—P1—Ni1—O2	−43.10 (7)
C32—C27—C28—C29	−1.6 (3)	C1—P1—Ni1—O2	−171.60 (7)
C26—C27—C28—C29	177.97 (17)	C13—P1—Ni1—N1	−33.36 (18)
O2—C28—C29—O1	8.5 (5)	C7—P1—Ni1—N1	−151.07 (18)
C27—C28—C29—O1	−171.9 (5)	C1—P1—Ni1—N1	80.43 (19)
O2—C28—C29—C30	−177.6 (2)	C13—P1—Ni1—S1	−105.85 (5)
C27—C28—C29—C30	2.0 (3)	C7—P1—Ni1—S1	136.44 (7)
O2—C28—C29—O1A	−8.3 (8)	C1—P1—Ni1—S1	7.95 (6)
C27—C28—C29—O1A	171.3 (8)	N2—C25—S1—Ni1	0.68 (15)
O1—C29—C30—C31	172.2 (6)	N3—C25—S1—Ni1	−176.12 (13)
O1A—C29—C30—C31	−169.0 (9)	N1—Ni1—S1—C25	1.20 (7)
C28—C29—C30—C31	−1.3 (4)	P1—Ni1—S1—C25	168.75 (6)
C29—C30—C31—C32	0.1 (5)	C30—C29—O1—C33	1.5 (9)
C30—C31—C32—C27	0.3 (5)	O1A—C29—O1—C33	−106 (4)
C28—C27—C32—C31	0.5 (4)	C28—C29—O1—C33	175.3 (5)
C26—C27—C32—C31	−179.1 (3)	C29—O1—C33—C34	−173.6 (8)
C27—C26—N1—N2	−179.63 (17)	O1—C29—O1A—C33A	73 (3)
C27—C26—N1—Ni1	7.3 (3)	C30—C29—O1A—C33A	−10.7 (19)
N3—C25—N2—N1	173.97 (14)	C28—C29—O1A—C33A	−179.3 (11)
S1—C25—N2—N1	−2.9 (2)	C29—O1A—C33A—C34A	−76.9 (19)

### Hydrogen-bond geometry (Å, º)

Cg7 is the centroid of the C27–C32 ring.

**Table:**

*D*—H···*A*	*D*—H	H···*A*	*D*···*A*	*D*—H···*A*
C12—H12···O1	0.93	2.46	3.314 (7)	154
C12—H12···O2	0.93	2.35	3.113 (2)	139
N3—H3*A*···N2^i^	0.87 (1)	2.22 (1)	3.0811 (19)	170 (2)
C33*A*—H33*D*···*Cg*7^ii^	0.97	2.79	3.279 (10)	112

Symmetry codes: (i) −*x*, −*y*+1, −*z*; (ii) −*x*+1, −*y*, −*z*+1.
