# Supplementary figures and images for: Crystal structure of [1-(3-eth­oxy-2-oxido­benzyl­idene-κO 2)-4-phenyl­thio­semicarbazidato-κ2 N 1,S](tri­phenylphosphane-κP)nickel(II)
Source: Acta Crystallogr E Crystallogr Commun. 2015 Nov 21;71(Pt 12):m230–1. doi: 10.1107/S2056989015021660 (PMC4719848; doi:10.1107/S2056989015021660)

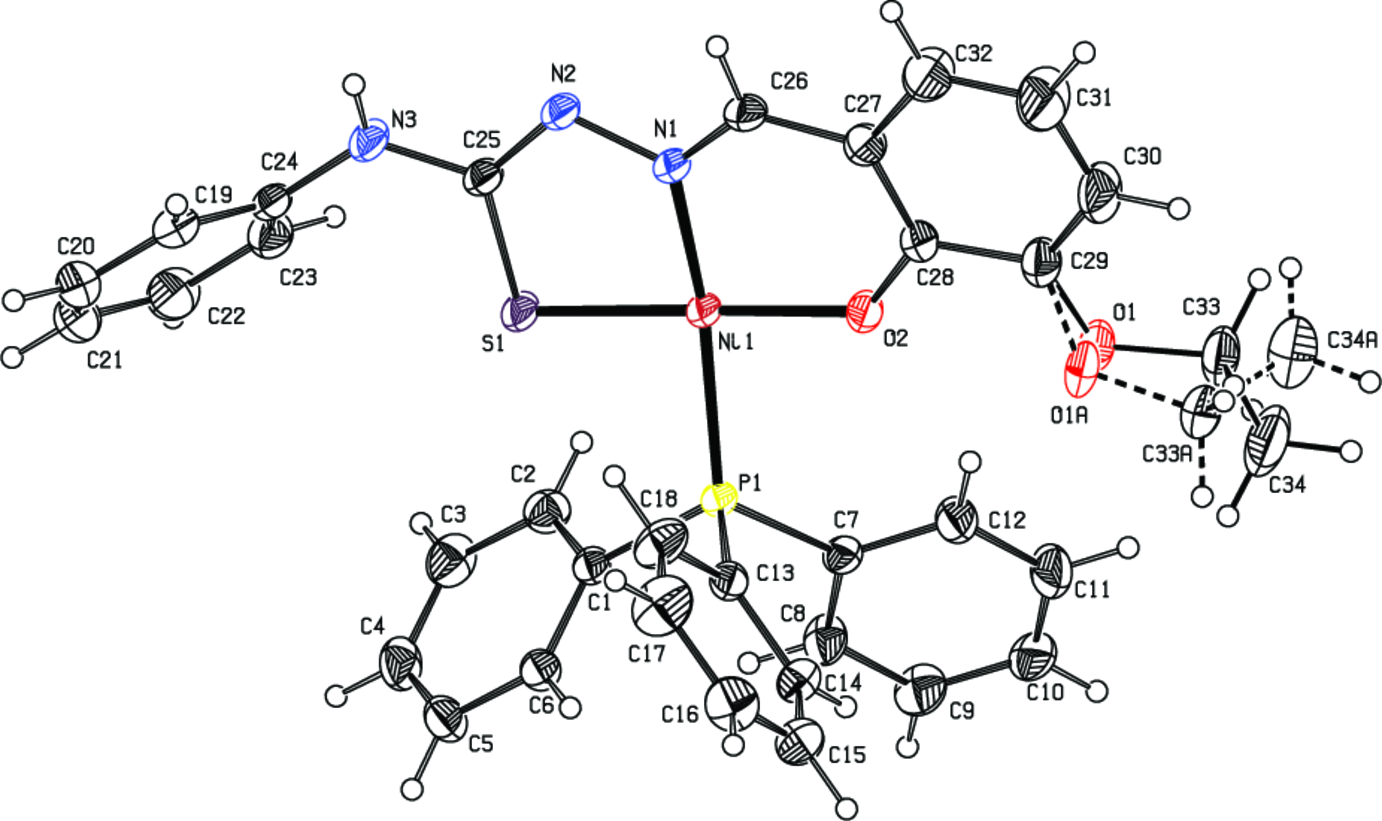

Supplement: Supplementary file 3 [file e-71-0m230-fig1.tif]

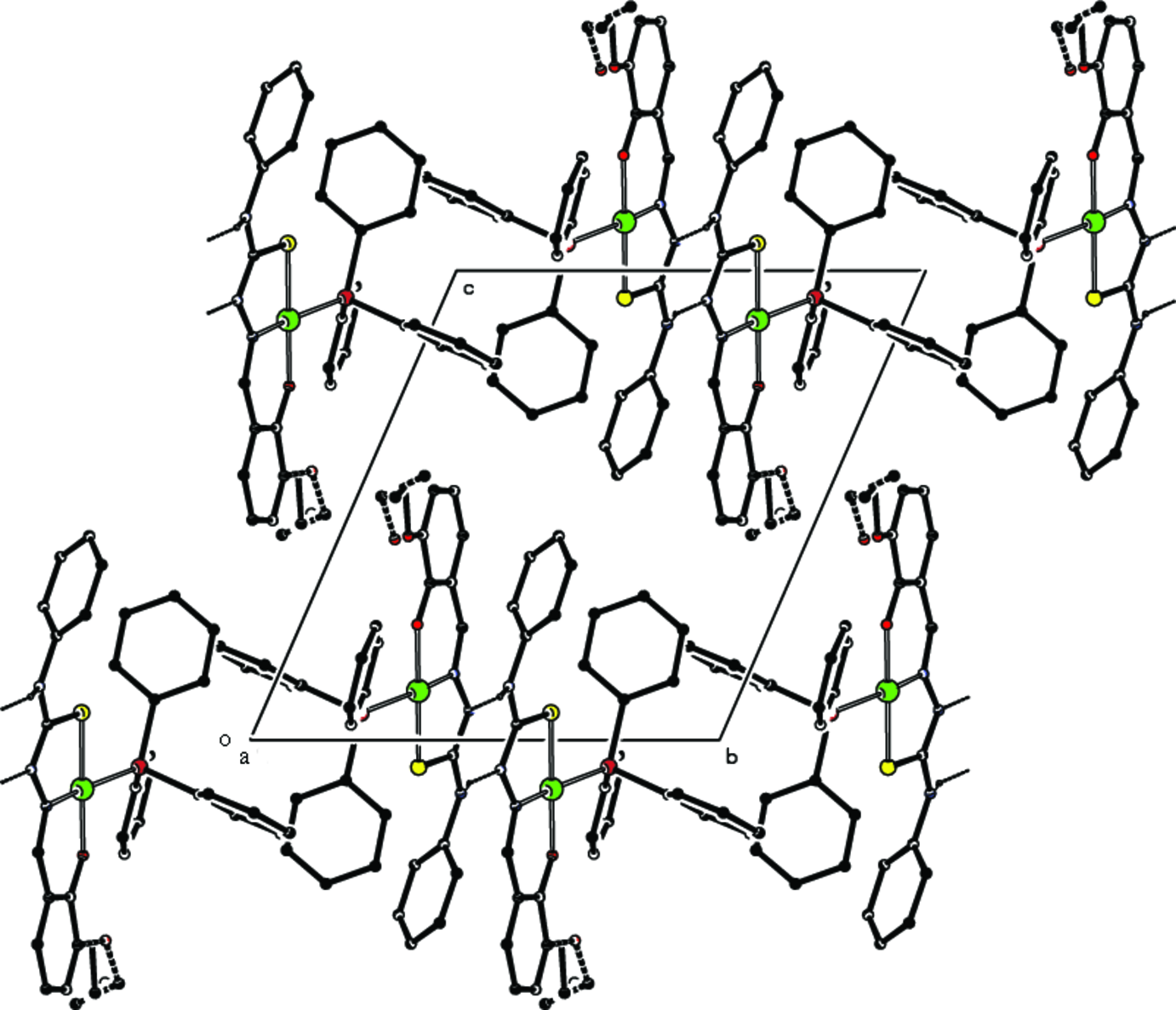

Supplement: Supplementary file 4 [file e-71-0m230-fig2.tif]

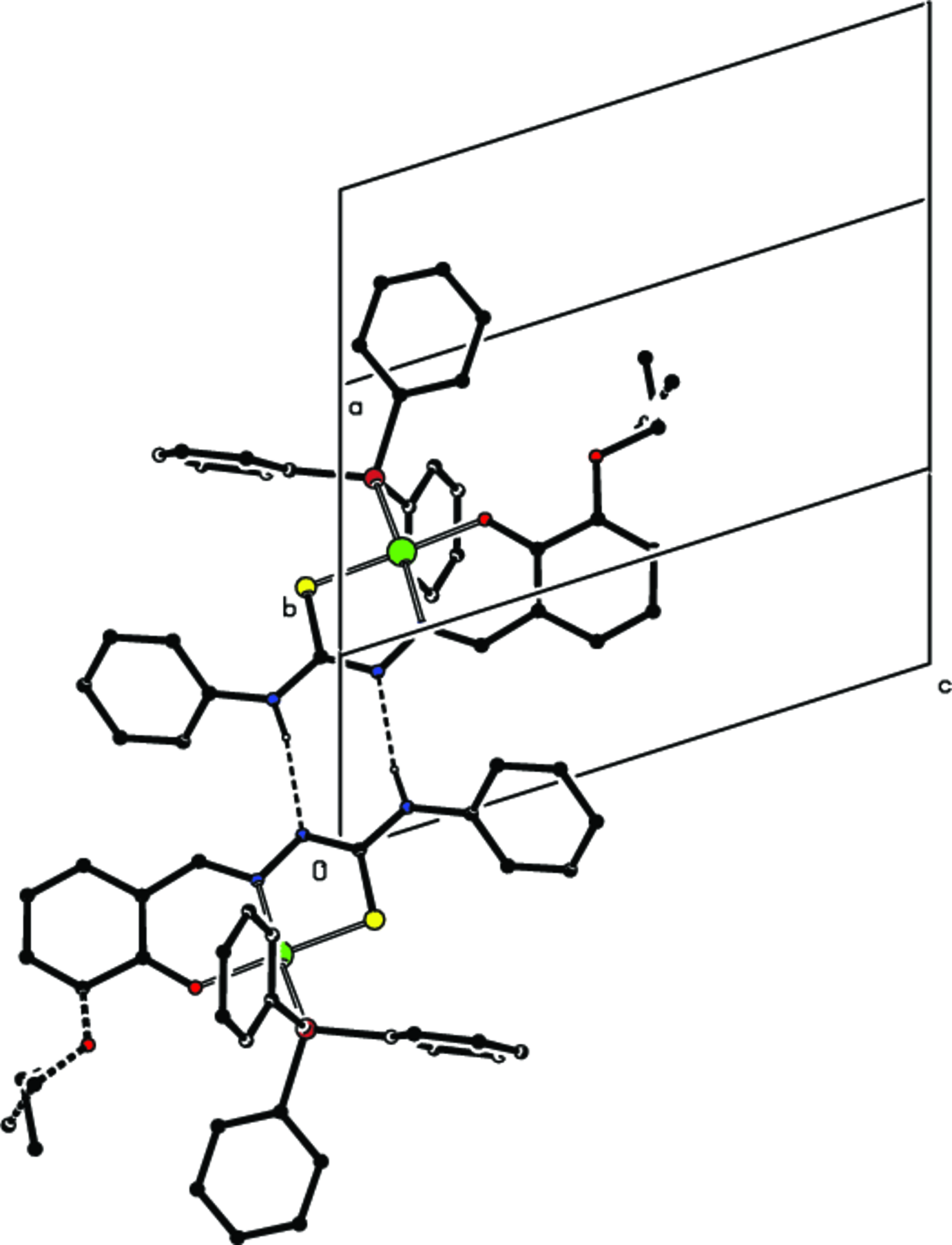

Supplement: Supplementary file 5 [file e-71-0m230-fig3.tif]
